# Vascular density normative data of radial peripapillary capillary plexus in healthy Caucasian subjects

**DOI:** 10.1007/s00417-024-06623-6

**Published:** 2024-08-30

**Authors:** Rita Serra, Antonio Pinna, Andrea Angius, Vincenzo Rallo, Michele Marongiu, Lenore Launer, Myriam Gorospe, David Schlessinger, Florence Coscas, Edoardo Fiorillo, Francesco Cucca

**Affiliations:** 1 Institute of Genetic and Biomedical Research, National Research Council, C/O S.S 554 Bivio Per Sestu Km 4,500. Cittadella Universitaria Di Cagliari. Monserrato, 09042 Cagliari, Italy; 2 Centre Ophtalmologique de L’Odeon, 113 Bd Saint Germain, 75006 Paris, France; 3 Present Address: Department of Medicine, Surgery and Pharmacy, Ophthalmology Unit, University of Sassari, Sassari, Italy; 4 Laboratory of Genetics and Genomics, National Institute On Aging, Baltimore, MD USA

**Keywords:** Caucasian population, Healthy subjects, Optical coherence tomography angiography, Radial peripapillary capillary plexus, Vascular density

## Abstract

**Purpose:**

To establish a normative database for vascular density (VD) of radial peripapillary capillary plexus (RPC) in healthy Caucasian subjects.

**Methods:**

633 healthy Caucasian subjects underwent a complete ophthalmological examination, including slit-lamp biomicroscopy, best corrected visual acuity measurement with Early Treatment Diabetic Retinopathy Study charts, intraocular pressure measurement, fundus examination, and macular and optic nerve head (ONH) structural optical coherence tomography (OCT). En-face 4.5 × 4.5 mm OCT angiography scans of the RPC plexus were recorded and VD values, automatically provided by the AngioAnalytics™ software, noted. We statistically estimated the impact of age and gender on RPC VD values using a linear mixed model.

**Results:**

560 subjects fully met inclusion criteria and, according to age, were stratified into 5 groups: 18–50 years (77), 51–60 years (160), 61–70 years (110), 71–80 years (132), and ≥ 81 years (81). Overall, mean RPC VD of the whole en-face image was 53.03 ± 4.27%. Age was significantly related to RPC VD values of whole en-face image (r = -0.454; p < 0.0001), which decreased with aging. The linear mixed model disclosed that age has a statistically significant effect on RPC VD values in whole en-face image (p = 0.0006). As age increases, RPC VD values decrease by 0.12 per year. Conversely, no significant gender-related differences were found in terms of RPC VD values of whole en-face image and each parapapillary quadrant analyzing all age group.

**Conclusions:**

Results show that RPC VD values in healthy Caucasian subjects decrease with aging. These data may be used to create a reference normative database useful for clinical use.

**Key messages:**

- *What is known*
  - Radial peripapillary capillary (RPC) plexus, consisting of long parallel capillaries with rare bifurcations and anastomosis and extending straight along the course of the retinal nerve fiber layer to the posterior pole, may be affected early in some optic nerve head (ONH) and retinal diseases.
- *What is new*
  - This study reports RPC vascular density (VD) values, automatically measured on optical coherence tomography angiography, in healthy Caucasian subjects, demonstrating that age is negatively related to RPC VD values.
  - Results show that RPC VD values in healthy Caucasian subjects decrease with aging. These data may be used to create a reference normative database useful for clinical use.

## Introduction

Radial peripapillary capillary (RPC) plexus was firstly described by Michaelson et. al in 1954 as a single vascular layer located at the level of the retinal nerve fiber layer (RNFL), surrounding the optic nerve head (ONH) [1]. Thereafter, in the 1960s, some authors suggested that RPC plexus consisted of long parallel capillaries with rare bifurcations and anastomosis, extending straight along the course of the RNFL to the posterior pole [2, 3].

Although fluorescein angiography still represents the gold standard for retinal and ONH vascular assessment, traditional angiographic techniques do not allow RPC visualization.

Optical coherence tomography angiography (OCTA), a relatively new retinal imaging technique, has now made it possible the detailed in vivo visualization of the RPC plexus, revolutionizing the imaging assessment of retinal and ONH vasculature [4]. OCTA provides high resolution images of the posterior pole vasculature, in few seconds and with no dye use [5].

Recent research has shown that the RPC plexus may be affected early in some ONH and retinal diseases. Some reports assessing ONH color-coded perfusion maps (grid-based flow density) have revealed that vascular density (VD) values in the nine peripapillary sectors are significantly reduced in diseases with a common vascular pathogenesis, such as glaucoma, retinal vein occlusion, and proliferative diabetic retinopathy [2, 6, 7].

The study of RPC plexus represents a hot topic in current retinal research, but data on RPC VD of healthy subjects are still limited [8–11]. To the best of our knowledge, no data have already been reported for the Italian population. The present study was undertaken to assess RPC VD values in healthy Caucasian subjects, to provide an age- and gender-related reference normative database for clinical and comparative use.

## Methods

This was a retrospective, observational study on healthy Italian subjects examined at the Genetic and Biomedical Research Institute of the National Research Council (CNR, Italy), Lanusei, Italy, between September 2019 and July 2020.

The present investigation was conducted in compliance with the tenets of the Declaration of Helsinki for research involving human subjects and all participants gave written informed consent to study protocols, which were approved by the Regional Ethical Committee (protocol no. 2171/CE, Italy).

The inclusion criteria were no history or clinical evidence of any retinal and ONH disease (i.e., glaucoma, posterior uveitis, retinal vein or artery occlusion, etc.) potentially confounding image interpretation, intraocular pressure (IOP) < 21 mm Hg, no chronic systemic or topical corticosteroid use, best correct visual acuity (BCVA) > 20/40, and refractive error between -6 and + 3 diopters.

Poor quality ONH images (signal strength index < 55) were excluded to limit potential bias related to the presence of motion artifacts, which may affect qualitative and quantitative analysis.

According to age, the study subjects were divided into 5 groups; group 1: 18–50 years, group 2: 51–60 years, group 3: 61–70 years, group 4: 71–80 age years, and group 5: ≥ 81 years.

All subjects underwent a complete ophthalmological examination, including BCVA measured using Early Treatment Diabetic Retinopathy Study charts, IOP measurement, fundus examination, macular and ONH structural OCT. OCTA scans of the ONH were performed using the AngioVue disc mode, which automatically segments the ONH into four layers: ONH, vitreous, RPC plexus, and choroid. We assessed 4.5 × 4.5 mm OCTA scans of the RPC plexus, extending from the inner limiting membrane (ILM) to 100 µm under the ILM, which we set as the lower boundary [7].

**Fig. 1:**
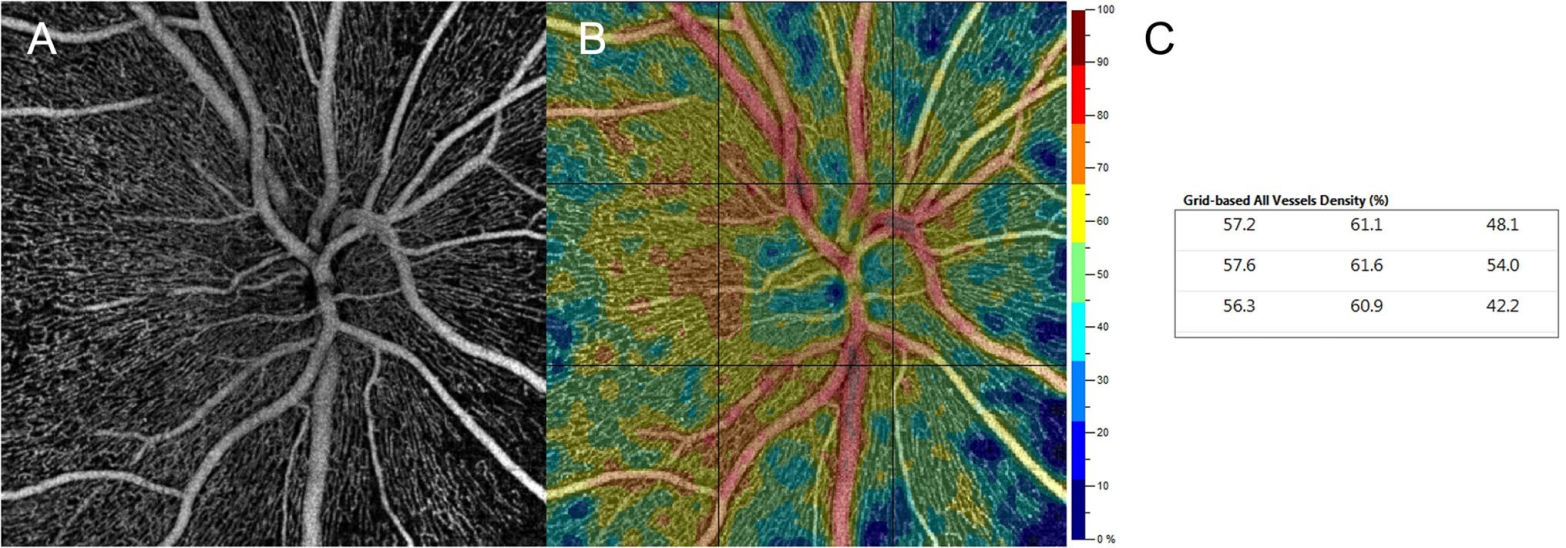
**Sample image of Optical Coherence Tomography Angiography (OCTA) of the radial peripapillary capillary (RPC) plexus. **RPC en-face angiogram (**A**), corresponding color-coded perfusion map (**B**), and grid-based vessels density (VD; **C**) in a 38-year-old healthy subject

The correct segmentation and quality of all ONH scans were carefully assessed by a retinal specialist (R.S.). VD values of RPC plexus were recorded automatically by the AngioAnalytics software embedded in the OCTA device. Similarly, we analyzed the color-coded perfusion maps generated by the flow density map software AngioAnalytics, which provide VD values of the nine different peripapillary sectors (grid-based flow density) of the entire ONH scan (Fig. 1).

For statistical analysis, only right eyes were considered. Descriptive analysis results are described as numbers and percentages for qualitative variables and as means ± standard deviation (SD) for quantitative variables. After testing normality data distribution, Student *t* test and Pearson test were used, when appropriate. Linear regression analysis was also performed. One-way ANOVA and pairwise Tukey–Kramer tests were performed to assess a possible correlation between RPC VD values of whole en-face image and age. A linear mixed-effects model fit by the restricted maximum likelihood (REML) approach allowed to estimate the random coefficients regression between age and RPC VD values within each age group to obtain an overall unbiased effect of age on RPC VD values. Age covariate was set as the fixed effect and the intercept random across age groups as random effect. Model was estimated using gamlj_mixed function from GAMLj3 package** [**https://github.com/gamlj/gamlj**]**. A p value < 0.05 was considered statistically significant. The study data were analyzed using the Statistical Package for Social Sciences version 20.0 for Mac (IBM, Chicago, IL, USA) and Jamovi statistical software program (The jamovi project, Sydney, Australia; Version 2.4.7; https://www.jamovi.org/**).**

## Results

The images of 633 right eyes of 633 healthy subjects were analyzed. Seventy-three eyes were excluded because of poor quality OCTA images. The remaining 560 eyes of 560 subjects (241 men, 319 women) met inclusion criteria and were considered for further analysis. This cohort was representative of the general population for % of gender and age distribution.

Overall, mean age was 64.79 ± 14.16 years and mean RPC VD of the whole en-face image was 53.03 ± 4.27%. Mean age was 64.74 ± 14.16 years in the male group and 64.68 ± 14.16 years in the female group, a not statistically significant difference. Mean RPC VD of the whole en-face image was 51.89 ± 4.26% in men and 53.04 ± 4.2% in women, respectively, again a not statistically significant difference. According to age, subjects were divided into 5 groups: 18–50 years (77), 51–60 years (160), 61–70 years (110), 71–80 years (132), and ≥ 81 years (81). No statistically significant gender-related differences were found in terms of RPC VD of en-face image in all age groups (Table 1).

**Table 1:** Comparison of radial peripapillary capillary (RPC) density values of whole en-face image between female and male groups. Variables are presented as mean ± standard deviation (SD)

Age groups	Eyes n	Men	Women
< 50 years	77	55.67 ± 3.03	55.64 + 3.06
Age = 51–60 years	160	54.51 + 3.30	54.66 + 3.27
Age = 61–70 years	110	52.38 + 4.01	52.55 + 3.99
Age = 71–80 years	132	51.39 + 4.54	51.33 + 4.52
Age > 81 years	81	50.15 + 3.43	50.48 + 3.14

**Fig. 2:**
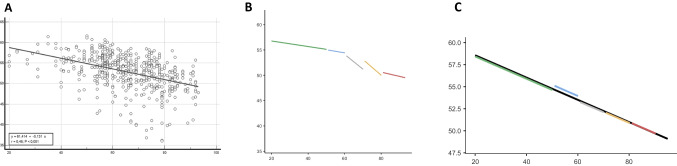
**A:** Linear regression for radial peripapillary capillary plexus (RPC) vascular density (VD) of whole en-face image. **B:** Scatterplot depicting the age groups classification (linear model), the points represent scores of RPC VD values given the age; **C:** Plot of the fixed and random effects together (linear mixed model). X axis: age; Y: RPC VD; Age groups: green: <50; blue: 51-60; grey: 61-70, yellow: 71-80, red> 81

RPC VD values of the whole en-face image was 52.97 ± 4.17%, a result significantly related to increasing age (r = -0.454; p < 0.0001). Likewise, RPC VD values of each parapapillary quadrant were significantly age-related. Specifically, young subjects showed higher values of RPC VD in whole en-face image and also in all the peripapillary sectors, when compared with older subjects (all p < 0.0001). Linear regression models indicated that RPC VD values of whole en-face image decreased significantly with increasing age (p < 0.0001). ANOVA and pairwise Tukey–Kramer tests showed a decreasing density with ageing (p < 0.001) (Fig. 2).

RPC VD values in the five age groups are summarized in Table 2. The highest VD belonged to the supero-middle and infero-middle zones, as expected, due to the anatomical orientation of the retinal vascular bundles.

**Table 2:** Radial peripapillary capillary (RPC) density values in 560 healthy subjects. First column includes en-face image and peripapillary sectors. Variables are presented as mean ± standard deviation (SD)

Age groups	< 50	51–60	61–70	71–80	> 81
Eyes n	77	160	110	132	81
En-face image	55.67 ± 3.03	54.66 ± 3.27	52.55 ± 3.88	51.38 ± 4.50	50.15 ± 3.43
Supero-temporal	54.90 ± 5.94	53.40 ± 6.30	51.69 ± 6.51	49.90 ± 7.15	49.03 ± 7.64
Supero-middle	58.15 ± 4.99	56.97 ± 5.06	54.16 ± 6.22	53.71 ± 6.56	51.83 ± 5.58
Supero-nasal	53.79 ± 6.58	52.84 ± 5.77	50.42 ± 6.72	49.03 ± 7.88	47.92 ± 6.50
Intermediate-temporal	53.90 ± 4.77	52.75 ± 5.24	51.67 ± 5.89	50.50 ± 6.04	49.64 ± 5.82
Intermediate-nasal	53.79 ± 5.19	52.93 ± 4.83	50.92 ± 5.11	49.86 ± 5.47	48.61 ± 5.98
Inferior-temporal	53.57 ± 7.82	52.19 ± 7.94	50.76 ± 7.63	49.57 ± 7.93	48.05 ± 7.90
Inferior-middle	59.89 ± 4.57	59.44 ± 5.69	57.30 ± 6.13	55.70 ± 6.14	53.25 ± 5.68
Inferior-nasal	53.25 ± 6.92	52.23 ± 6.91	49.78 ± 7.49	48.75 ± 7.91	47.87 ± 7.54

The linear mixed model disclosed that age has a statistically significant effect on RPC VD values in whole en-face image (p = 0.0006). As age increases, RPC VD values decrease by 0.12 per year (Table 3).

**Table 3:** Fixed effects parameters estimate. Variables tested (Names), variable’s estimates (Estimate), Standard Error (SE), 95% Confidence Interval (Lower and Upper), p-value (p)

			95% Confidence Interval		
Names	Estimate	SE	Lower	Upper	p
Intercept	61.08	0.97	59.18	62.98	1.03E-09
Age	-0.12	0.01	-0.15	-0.09	1.05E-04

Furthermore, linear regression analysis revealed that a model where each age group is allowed to express a different regression line would not fit the data better than a model with only one regression line, fixed for every age group (Fig. 2). Both the low variance of the intercept (0.14) and the intraclass correlation coefficient (ICC = 0.01) determined that the observations within age groups are no more similar than observations from different clusters. In Fig. 2, we calculated the linear and mixed linear model using the age groups older than 50 years, which are numerically more consistent and most representative of the changes in RPC VD values.

## Discussion

We investigated the VD of the RPC plexus to provide VD mapping data in 560 healthy Caucasian subjects. Our analysis revealed that mean RPC VD of the whole image was 53.03 ± 4.27%, with the supero-middle and infero-middle quadrants showing the highest VD values.

A similar result (RPC VD = 53.8%) was described by Zhu et al. [8] in a cohort of 1487 healthy Chinese subjects (613 men, 874 women; mean age: 48.8 ± 15.4 years). However, the subjects included in our study were approximately 20 years older than those assessed by Zhu et al. [8] (mean age: 64.79 ± 14.16 vs. 48.8 ± 15.4 years). Conversely, our whole en-face RPC VD are higher than those reported by Pinhas and coworkers [9], who evaluated a cohort of 133 healthy subjects (59 men, 74 women, mean age 41.5 years) much younger than ours.

Aging seems to play a critical role in anatomical changes in the retina and ONH, mainly due to oxidative stress-induced damage. Physiologically, during aging, reactive oxidative species (ROS) levels increase and antioxidants decline, thus inducing attenuation of ONH and retinal parameters [11–13].

Owing to its elevated metabolic activity, the retina is one of the highest oxygen-consuming human tissues and strongly susceptible to oxidative stress-induces damage. However, Polidori et al. [14], evaluating a Mediterranean population, found that elderly and young people, living in the same geographic area, exhibited similar levels of oxidative stress, suggesting that age-related changes may occur at a slower pace in such populations [12].

Our results show that younger subjects have significantly higher values of RPC VD in whole en-face image and also in all peripapillary sectors, when compared with older individuals. This result is probably dependent on atherosclerosis, one of the most important degenerative disorders, characterized by thickening and narrowing of the arterial walls [15]. Initially, atherosclerosis causes loss of elasticity of vessel walls; then, with aging, the narrowing of capillaries and slowing of blood flow leads to VD value decrease.

Interestingly, recent research has shown that Glucose-6-Phosphate-Dehydrogenase (G6PD)-deficient individuals have higher VD values of RPC plexus than age-matched controls [7]. G6PD deficiency is an inherited enzymatic disorder affecting more than 500 million people worldwide [16]. This condition, which is common in Mediterranean populations, appears to protect against the occurrence of vascular disorders, including retinal vein occlusion (RVO) and non-arteritic anterior ischemic optic neuropathy (NA-AION) [17–19].

Why and how G6PD deficiency may offer protection against some vascular disorders is still a matter of debate. Experimental evidence indicates that G6PD-deficient cells show reduced cholesterol synthesis and esterification, which may account for the decreased cholesterol accumulation in the arteries of these individuals and, consequently, for the lower risk of atherosclerotic vascular disorders, including RVO and NA-AION [7, 20].

Furthermore, the low oxidative levels detected in G6PD-deficient individuals appear to offer protection against age-related biochemical changes, especially in the retina. G6PD-deficient subjects exhibit a better capillary network, (higher RPC VD), due to decreased NADPH production and NOX2 activity in vessel walls [7].

Animal models have revealed that endothelial Nox2 is strongly associated to the age-related capillary rarefaction observed in the nervous system, due to increased ROS production [7]. It is possible that G6PD-deficient subjects, showing lower oxidative stress levels with decreased NO and GSH production, may have higher RPC VD values than age-matched controls.

In this study, we evaluated the whole en-face image and each parapapillary quadrant to establish whether, or not, there is some gender-related correlation, but failed to find any statistical difference.

The role of sex hormones in vascular pathologies is still unclear. Laser speckle flowgraphy disclosed significant gender-related differences in blood flow velocity in ONH [21, 22]. It has been postulated that sex hormones are implicated in vascular pathophysiology and ocular hemodynamic regulation. Estrogen levels promote vascular smooth muscle relaxation and influence flow speed and pulsatility index in ocular vessels, with protective effects against ocular vasculopathies [23]. Such effects, no longer evident after menopause [23], could explain the absence of statistically significant gender-related differences in RPC VD, since our study included mainly women aged > 50 years.

Our study has several limitations. First, the size of the younger group was relatively small, although the total numerosity was comparable to other studies. Second, we did not perform axial length correction because vessel density was calculated area-to-area ratio (RPC pixel area divided by ROI pixel area) [9]. Third, there is an intrinsic limitation due to the OCTA software, which may introduce artifacts. Although OCTA slabs of RPC layer should not present projection artifacts, other errors, including loss of detail, doubling of vessels, and stretching defects, may affect RPC OCTA images [10]. To limit the bias due to such errors, only high-quality OCTA images were considered, and the correct segmentation was carefully assessed by an expert retinal specialist. Fourth, in this study we analyzed only right eyes. Therefore, we do not know whether, or not, there are any differences in RPC VD between eyes. This topic will be the subject of future research. Last but not least, RPC VD values reported in the present study were obtained using the AngioVue disc mode embedded in the OCTA device. This limitation implies that our results can ideally be used only for comparisons with other data obtained on the same platform.

In conclusion, we analyzed RPC VD data of whole en-face image and each parapapillary quadrant in a cohort of healthy Caucasian subjects. We found that there is an inverse correlation between RPC VD values and increasing age. As far as we know, this is the first study providing RPC VD normative data for a Caucasian population, which may be useful as a reference database for clinical use. Future larger, multicenter studies on this topic are warranted.
